# Comparison of Exogenous Ketone Administration Versus Dietary Carbohydrate Restriction on Myocardial Glucose Suppression: A Crossover Clinical Trial

**DOI:** 10.2967/jnumed.121.262734

**Published:** 2022-05

**Authors:** Senthil Selvaraj, Kenneth B. Margulies, Supritha Dugyala, Erin Schubert, Ann Tierney, Zoltan Arany, Daniel A. Pryma, Svati H. Shah, J. Eduardo Rame, Daniel P. Kelly, Paco E. Bravo

**Affiliations:** 1 Division of Cardiology, Department of Medicine, Perelman School of Medicine of the University of Pennsylvania, Philadelphia, Pennsylvania;; 2 Division of Nuclear Medicine, Department of Radiology, Perelman School of Medicine of the University of Pennsylvania, Philadelphia, Pennsylvania;; 3 Department of Biostatistics, Perelman School of Medicine of the University of Pennsylvania, Philadelphia, Pennsylvania;; 4 Division of Cardiology, Department of Medicine, Duke Molecular Physiology Institute, Duke University School of Medicine, Durham, North Carolina; and; 5 Department of Cardiology, Thomas Jefferson University, Abington, Pennsylvania

**Keywords:** ketogenic diet, ketone ester, FDG, PET, myocardial glucose uptake

## Abstract

The ketogenic diet (KD) is the standard of care to achieve myocardial glucose suppression (MGS) for assessing inflammation using ^18^F-FDG PET. However, failure to suppress physiologic glucose uptake remains a significant diagnostic barrier. Although extending the duration of KD may be effective, exogenously delivered ketones may provide a convenient, reliable, and same-day alternative. The aims of our study were to determine whether exogenous ketone administration is noninferior to the KD to achieve MGS and whether serum β-hydroxybutyrate (BHB) levels can predict MGS. **Methods:** KEETO-CROSS (Ketogenic Endogenous versus Exogenous Therapies for myoCaRdial glucOse SuppresSion) is a crossover, noninferiority trial of the KD (endogenous ketosis) versus ketone ester ([KE] exogenous ketosis) drink. Twenty healthy participants were enrolled into 3 arms: weight-based KE drink, 24-h KD, and 72-h KD (*n* = 18 completed all arms). The primary outcome was achievement of complete MGS on PET (noninferiority margin 5%). The area under receiver-operating-characteristics (AUROC) of endogenous BHB levels (analyzed in a laboratory and by point-of-care device) for predicting MGS was analyzed in 37 scans completed on the KD. **Results:** The mean age was 30 ± 7 y, 50% were women, and 45% were nonwhite. The median achieved BHB levels (mmol/L) were 3.82 (25th–75th percentile, 2.55–4.97) (KE drink), 0.77 (25th–75th percentile, 0.58–1.02) (25th–75th percentile, 24-h KD), and 1.30 (25th–75th percentile, 0.80–2.24) (72-h KD). The primary outcome was achieved in 44% (KE drink), 78% (24-h KD), and 83% (72-h KD) of participants (noninferiority *P* = 0.97 and 0.98 for KE vs. 24-h and 72-h KD). Endogenous BHB levels robustly predicted MGS (AUROC, 0.88; 95% CI 0.71, 1.00). A BHB of 0.58 or more correctly classified 92% of scans. A point-of-care device provided comparable predictive value. **Conclusion:** In healthy volunteers, KE was inferior to KD for achieving MGS. Serum BHB is a highly predictive biomarker for MGS and can be clinically implemented upstream of ^18^F-FDG PET, with rapid facilitation by point-of-care testing, to reduce false-positive scans.

## Visual Abstract

**Figure:**
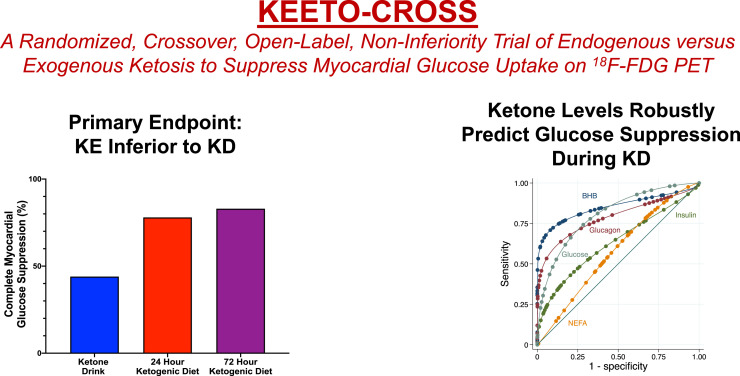

Detecting inflammation is clinically relevant for diagnosing several cardiovascular diseases, though it remains challenging to achieve by current techniques. Noninvasive diagnosis of a growing number of inflammatory pathologies, as well as malignant cardiac masses, relies on visualizing glucose uptake by abnormal cells using ^18^F FDG PET. However, because normal myocardium can also use glucose (*1*), distinguishing physiologic from pathologic uptake can be particularly problematic and remains the Achilles heel of using ^18^F-FDG PET for such diagnostic testing (*2*).

Short-term dietary modification through a low-carbohydrate, high-fat ketogenic diet (KD) to suppress physiologic glucose uptake is the standard of care for evaluating myocardial inflammation using radiolabeled markers of glucose utilization. The KD accomplishes this metabolic switch by reducing insulin-dependent myocardial glucose uptake, while inflammatory cells continue to consume glucose through non–insulin-dependent glucose transporters. However, myocardial ^18^F-FDG suppression rates remain suboptimal, and retrospective data suggest myocardial glucose suppression (MGS) is achieved in 81%–84% of subjects even after strict, highly supervised dietary restrictions (*3**,**4*). Nondiagnostic and false-positive scans lead to misdiagnosis, inappropriate immunotherapy, repeated scans with excess radiation exposure, and unnecessary costs to the patient and health-care system.

One strategy to improve appropriate MGS is to increase the length of dietary modification (*5*). However, adherence to the KD may not always be feasible, is often challenging, and requires advanced patient preparation. The negative correlation between carbohydrate and ketone use by the heart has been known for years (*6*), and exogenous ketones significantly reduce myocardial glucose uptake through substrate competition and inhibition of intracellular glucose phosphorylation (*7*). A ketone ester (KE) compound can rapidly and safely achieve high levels of ketosis and may efficiently and effectively prepare patients for same-day evaluation of myocardial inflammation by ^18^F-FDG PET (*8*). However, no studies have directly compared a KE with a KD strategy for achieving MGS. Additionally, given the inverse relationship between ketosis and MGS, ketone biomarkers may logically predict MGS. Despite promising data, neither hypothesis has been evaluated.

Ketogenic Endogenous versus Exogenous Therapies for myoCaRdial glucOse SuppresSion (KEETO-CROSS) is a crossover, noninferiority trial of an MGS strategy comparing KE with the gold- standard 24-h KD, as well as 72-h KD, in participants free of cardiovascular disease. We sought to determine whether a nutritional ketosis strategy would provide noninferior diagnostic value compared with short-term KD to achieve MGS on ^18^F-FDG PET. We secondarily assessed the value of serum ketone levels (β-hydroxybutyrate [BHB]) as an upstream biomarker of MGS on ^18^F-FDG PET to reduce false-positive and nondiagnostic scans.

## MATERIALS AND METHODS

### KEETO-CROSS Study Design

We conducted a crossover, open-label, noninferiority trial comparing exogenous ketosis (KE) with endogenous ketosis (KD) (NCT04275453). Participants were randomly assigned to the KE arm (1 visit) or KD arm (including 2 visits occurring at 24 and 72 h) with at least a 1-wk washout period in between arms. Participants aged 18–60 y were enrolled at the University of Pennsylvania between January 2020 and January 2021. To ensure that any myocardial glucose uptake would be physiologic rather than indicative of underlying pathology, we excluded individuals with any reported history of cardiovascular disease (including hypertension, hyperlipidemia, and diabetes mellitus). We also excluded pregnant and breast-feeding women. Recruitment was continued until a total of 18 participants attended all 3 visits (KE, 24-h KD, and 72-h KD) to adequately power the trial. The CONSORT diagram for KEETO-CROSS is depicted in Supplemental Figure 1 (supplemental materials are available at http://jnm.snmjournals.org). The study was approved by an institutional review board and informed consent was obtained.

### Patient Preparation Methods

#### KE Arm

**FIGURE 1.**
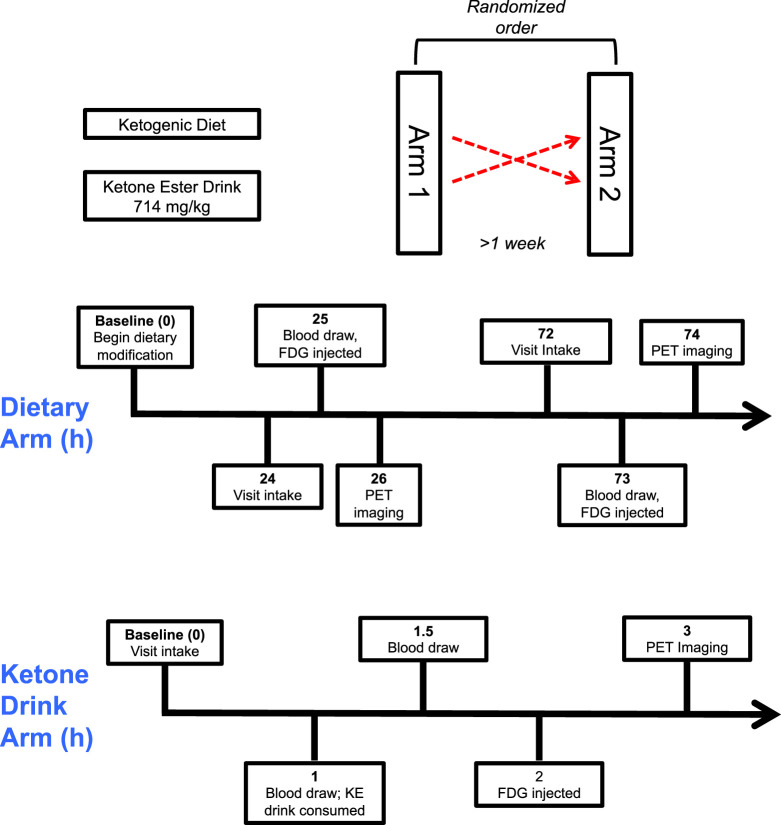
KEETO-CROSS study design. Participants were assigned in random order to KE and 24/72-h KD arms, and after at least a 1-wk washout period, participants returned for remaining arm. Visit time points in hours depicted. KD = ketogenic diet; KE = ketone ester.

Participants in the KE arm were permitted to eat a regular diet during the day before their study visit and started fasting at least by midnight before ^18^F-FDG PET (Fig. 1). During the visit, participants received weight-based dosing (714 mg/kg) of (*R*)-3-hydroxybutyl (*R*)-3-hydroxybutyrate (H.V.M.N.), which has been extensively studied for the purposes of achieving ketosis (*8*–*10*). Briefly, this KE undergoes rapid enzymatic hydrolysis ultimately to form BHB (and other ketone bodies), achieving high BHB levels in less than 1 h with a short compound half-life (0.8–3.1 h). This high dose was selected to engender robust ketosis and substrate competition. Participants were injected with ^18^F-FDG at 1 h after KE ingestion because the peak ketotic effect of the KE occurs around this time.

#### KD Arm

During the KD arm, participants presented for 2 visits: one after 24 h of KD and then again after 72 h of KD (Fig. 1). The day before the 24-h KD visit, participants began a low-carbohydrate, high-fat diet intended to achieve less than 20 g of carbohydrate intake per day (supplemental materials) and fasted from at least midnight until ^18^F-FDG PET scanning the next day. Thereafter, they continued the KD for 2 additional days, fasting again from at least midnight before the 72-h KD visit. A detailed dietary log was maintained by study participants during the dietary arm and reviewed by a nuclear cardiologist before ^18^F-FDG injection. All participants were deemed to have adequate dietary adherence by dietary log review.

### Laboratory Testing and Echocardiography

Details of laboratory testing (for BHB, insulin, glucagon, nonesterified fatty acid [NEFA], and glucose levels) and echocardiography are available in the supplemental materials.

### ^18^F-FDG PET Protocol and Study Outcomes

Participants underwent ^18^F-FDG PET using a PET/CT scanner (Ingenuity TF; Philips). At study visits, ^18^F-FDG (∼185 MBq [∼5.0 mCi]) was injected, and PET images of the chest were acquired 1 h later. Low-dose CT images were also acquired for attenuation-correction purposes and to aid in distinguishing ^18^F-FDG activity in the myocardium and blood pool on fused PET/CT images. Images were analyzed using MIM software by a board-certified nuclear cardiologist masked to participant characteristics. The primary outcome of the trial was complete MGS, defined by ^18^F-FDG activity in all segments of the myocardium lower than the blood pool (*2*). The secondary trial outcome was the ratio of the average myocardial to blood pool SUV in the septal and lateral walls.

### Statistical Analysis

Baseline characteristics were described using mean ± SD and medians and 25th–75th percentiles or percentages as appropriate for the levels of measurement and distributions of the variables. Biomarker levels were compared using nonparametric testing because they were right-skewed, and the false-discovery rate method was used for multiple testing correction.

We prespecified a modified-intention-to-treat analysis among participants who completed all 3 visits. A sample size of 18 participants provided 80% power to detect a 10% difference between the KE and 24-h KD group with a 5% noninferiority bound at an α-level of 5% and SD of paired differences of 0.36, assuming a correlation of 0.62 between studies. The primary (dichotomous) outcome of the trial was assessed using the exact noninferiority test of the difference between paired binomial proportions. Because noninferiority was only specified for the primary outcome, the secondary (continuous) outcome was analyzed using Wilcoxon signed-rank test because data were right-skewed. To limit multiple testing, the primary analysis compared KE and the 24-h KD (gold standard), whereas other comparisons are considered exploratory. To understand reasons for MGS failure with the KE drink, we used logistic regression to assess the relationship between log-transformed, post-KE drink biomarker levels with MGS, displaying the odds ratio per SD increase for ease of comparison between biomarkers.

In a prespecified analysis, we assessed ROCs of biomarkers using c-statistics to predict achievement of MGS, accounting for clustering at the participant level when applicable. For ease of comparison between biomarkers, c-statistics for insulin and glucose levels were displayed modeling the risk for MGS failure because these biomarkers are theoretically inversely related to MGS, whereas other biomarkers are directly related. We used continuous splines with 4 knots after confirming nonlinearity to model endogenous BHB levels and the myocardium to blood-pool SUV ratio. Logistic splines were also used to model the relationship between MGS and endogenous ketone levels. We further assessed the diagnostic value of point-of-care BHB values after the protocol modification. Bland–Altman plot and Spearman ρ were used to compare ketone levels by laboratory and point-of-care analysis. Analyses were performed separately for endogenous and exogenous ketone levels because each mode of ketosis reflects differing cardiovascular physiology and mechanisms of action (*1*).

Analyses were performed using STATA version 14 (STATA Corp.) and StatXact-12 (Cytel). For the primary outcome assessing noninferiority, a 1-sided *P* value of <0.05 was considered significant, whereas other analyses used a 2-sided *P* value of <0.05.

## RESULTS

The baseline characteristics of the 20 KEETO-CROSS heathy participants who completed 57 ^18^F-FDG PET scans are shown in Supplemental Table 1. The mean age was 30 ± 7 y, 50% were women, and 45% were nonwhite. The average duration of fasting (hours) before ^18^F-FDG injection was 15.9 ± 1.3 (KE drink arm), 16.7 ± 1.6 (24-h KD arm), and 15.2 ± 2.0 (72-h KD arm).

From a hemodynamic perspective, systolic blood pressure (119 ± 12 to 124 ± 11 mm Hg; *P* = 0.023) and heart rate (62 ± 12 to 71 ± 13 beats per minute; *P* < 0.001) increased significantly at approximately 30 min after consumption of the KE drink. Heart rate also showed a trend for increment (68 ± 13 and 76 ± 12 bpm; *P* = 0.14), whereas systolic blood pressure (117 ± 15 and 117 ± 11 mm Hg; *P* = 0.90) did not change with diet-induced ketosis at 24 and 72 h, respectively.

### Metabolite and Hormones Levels by Study Arm

**FIGURE 2.**
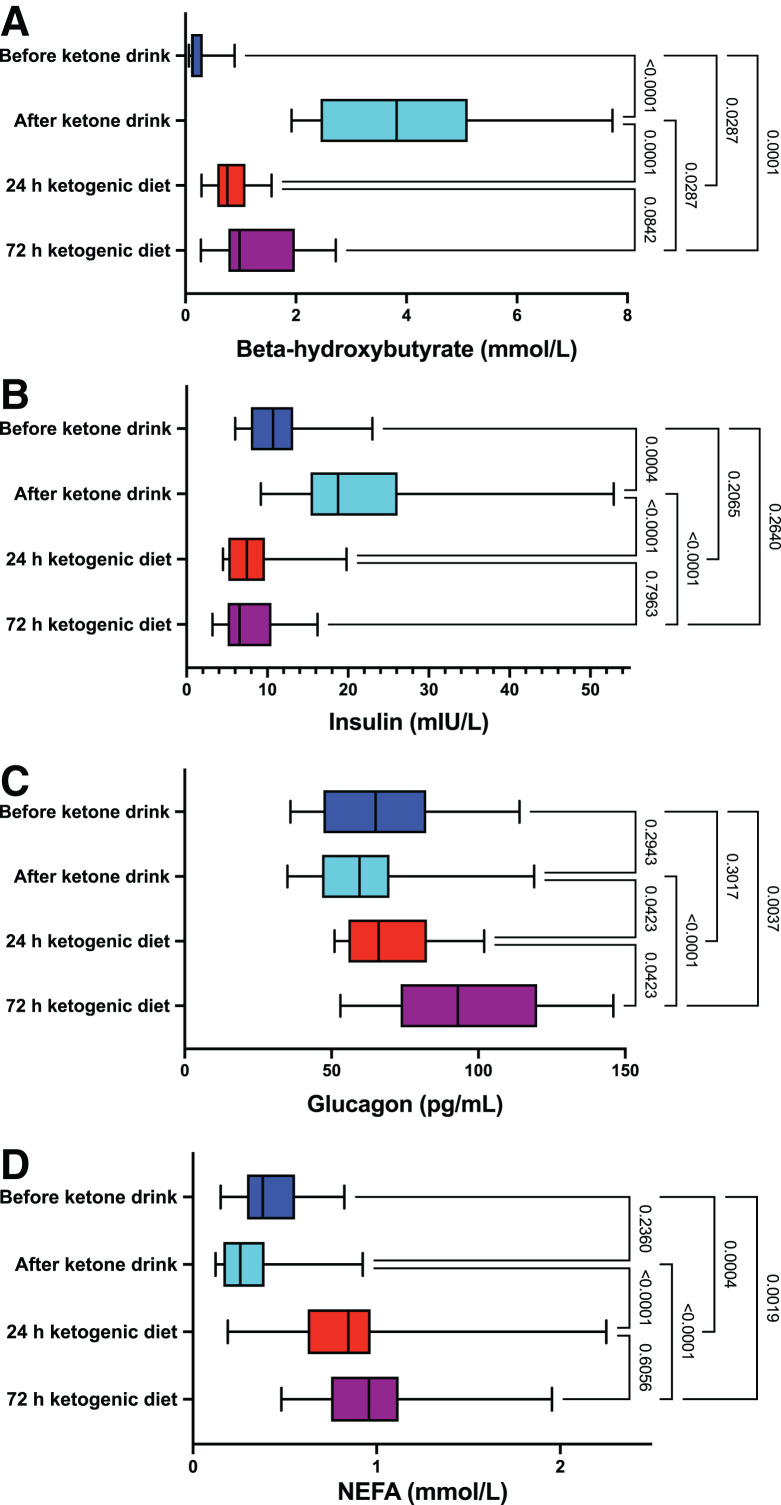
Biomarker levels by study arm. Box-and-whisker plots for ketone (A), insulin (B), glucagon (C), and NEFA (D) levels are displayed by study visit for the 18 participants included in the modified intention-to-treat analysis. Whiskers depict minimum and maximum values. *P* values corrected for multiple testing.

BHB, NEFA, insulin, and glucagon levels by study visit are displayed in Figure 2 for the 18 participants in the modified intention-to-treat analysis. BHB levels (presented as median and 25th–75th percentile) increased rapidly and significantly from immediately prior (median, 0.12; 25th–75th percentile, 0.10–0.26 mmol/L) to 30 min after KE drink ingestion (median, 3.82; 25th–75th percentile, 2.55–4.97 mmol/L, *P* < 0.001). Post-KE drink levels were significantly higher than that achieved by 24-h KD (median, 0.77; 25th–75th percentile, 0.58–1.02 mmol/L, *P* < 0.001) and 72-h KD (median, 1.30; 25th–75th percentile, 0.80–2.24 mmol/L, *P* = 0.029). A similar pattern was observed when comparing the post-KE drink insulin levels with other times points. Glucagon and NEFA levels were lower after the KE drink than for the 24- and 72-h KD arms (*P* < 0.05 for all comparisons), though they were not different from pre-KE drink levels (*P* > 0.20 for both comparisons). As expected, glucose levels were lower after the 24-KD and 72-KD groups compared with overnight fasting, reflected by the pre-KE drink levels (Supplemental Table 2).

### KEETO-CROSS Primary and Secondary Outcomes

**FIGURE 3.**
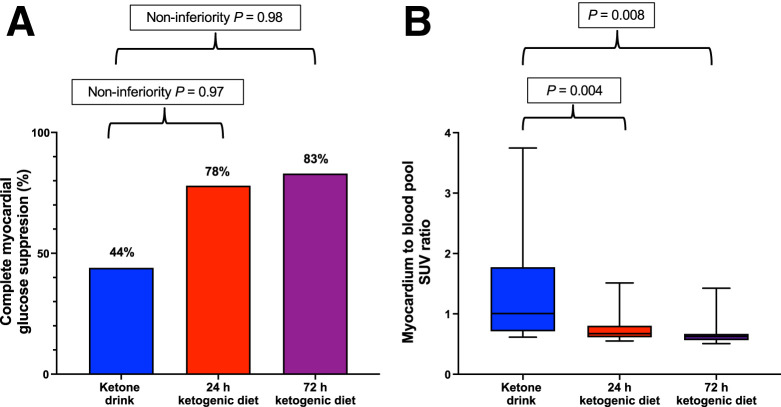
Primary and secondary endpoints of KEETO-CROSS study shown for the 18 participants who completed all study visits. (A) Percentages of primary endpoint achievement (complete myocardial glucose suppression). (B) Box-and-whisker plots of secondary outcome (average SUV of myocardium to blood pool).

Using the modified intention-to-treat protocol, we assessed the primary outcome (complete MGS) in the 18 participants who completed all 3 study visits (Fig. 3). Complete MGS was achieved in 8 of 18 (44%), 14 of 18 (78%), and 15 of 18 (83%) participants in the KE, 24-h KD, and 72-h KD arms, respectively. KE failed to meet the noninferiority bound compared with the KD arms (noninferiority *P* = 0.97 and 0.98 for KE vs. 24-h and 72-h KD, respectively).

Among the 18 participants completing all 3 visits, the secondary outcome (average SUV myocardium/blood pool, presented as median) was 1.01 (25th–75th percentile, 0.72–1.70), 0.67 (25th–75th percentile, 0.61–0.79), and 0.63 (25th–75th percentile, 0.56–0.66) in the KE, 24-h KD, and 72-h KD arms, respectively (*P* = 0.008 for KE vs. 24-h KD, and *P* = 0.004 for KE vs. 72-h KD) (Fig. 3).

Individual-level responses to MGS strategies are shown in Supplemental Figure 2. Two participants failed at least 1 dietary strategy and suppressed with the KE (Supplemental Fig. 3).

### Utility of Biomarkers to Predict Myocardial Glucose Suppression

**FIGURE 4.**
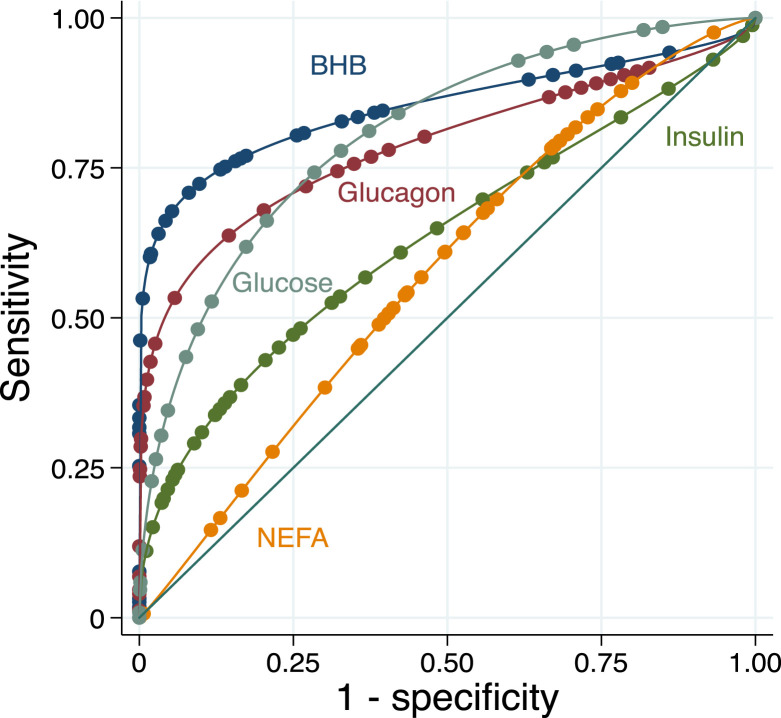
Receiver-operating-characteristic curve analysis for BHB, insulin, glucagon, NEFA, and glucose to predict myocardial glucose suppression in all participants during ketogenic diet.

Figure 4 shows area under the receiver-operating-characteristics curve (AUROC) for endogenous serum BHB levels to predict MGS in the KD arms (*n* = 37 scans). Serum BHB levels robustly predicted MGS (c-statistic 0.88, 95% CI 0.71–1.00). A threshold serum BHB level < 0.34 mmol/L correctly classified failure to achieve MGS, whereas BHB levels ≥ 1.09 mmol/L correctly classified MGS success, in these ^18^F-FDG PET scans. As a single threshold, using a BHB level ≥ 0.58 mmol/L for predicting MGS resulted in the highest correct classification of any value (92%), including 6 of 7 (86%) scans that were correctly classified as likely failure and 28 of 30 (93%) studies that were correctly classified as likely MGS based on BHB results. Glucose (c-statistic 0.78, 95% CI 0.61–0.95), glucagon (0.75, 95% CI 0.56–0.94), insulin (0.69, 95% CI 0.44–0.92), and NEFA (0.66, 95% CI 0.42–0.90) were less predictive of MGS, in comparison with BHB.

**FIGURE 5.**
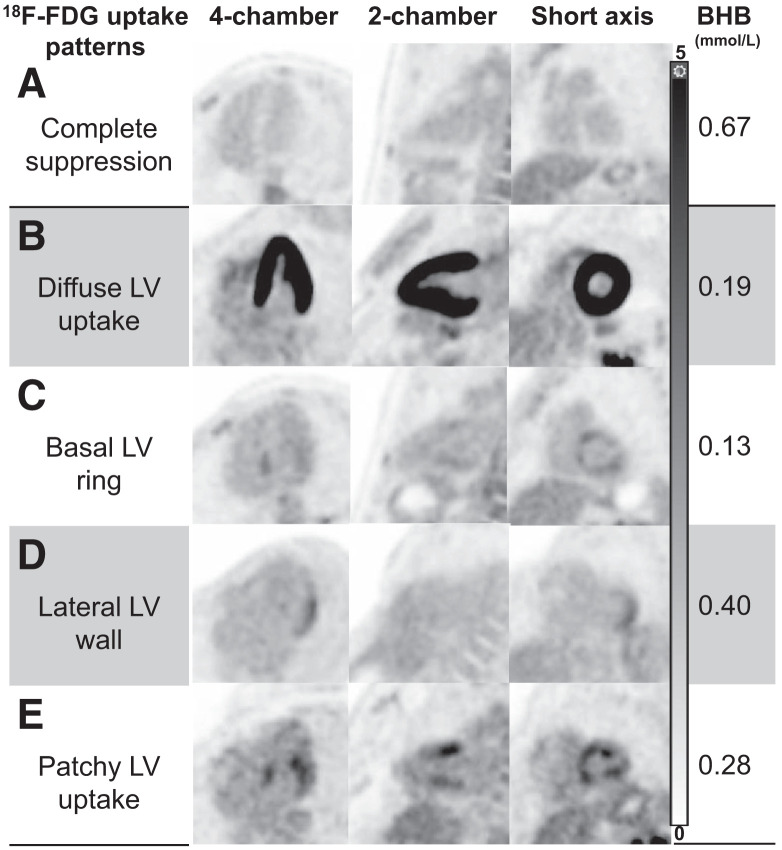
Myocardial ^18^F-FDG uptake patterns in healthy volunteers. Representative images (displayed using same window width 0–5) of most common myocardial ^18^F-FDG uptake patterns encountered in our healthy cohort, with their corresponding BHB levels. Patterns C–E can be potentially mistaken as myocardial inflammation; however, accompanying BHB levels should raise concern for incomplete suppression. LV = left ventricular.

**FIGURE 6.**
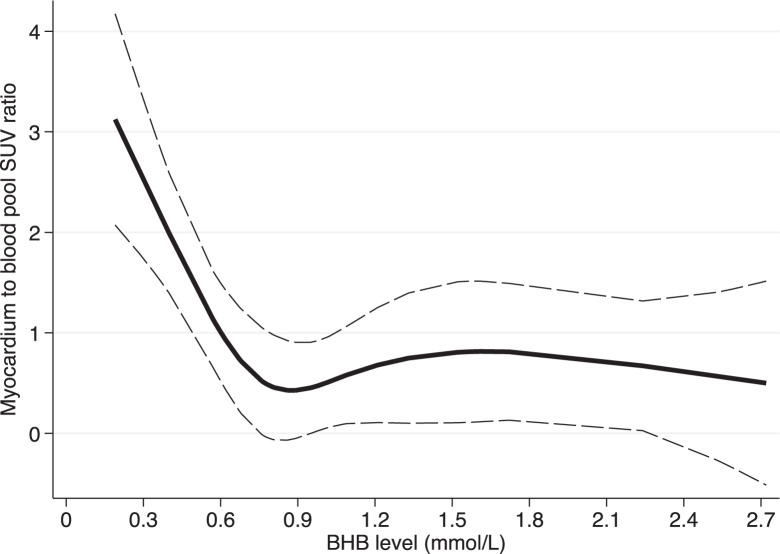
Relationship between endogenous ketone levels and myocardial glucose uptake. Continuous spline analysis with 4 knots depicts relationship between endogenous ketone levels (BHB) and secondary outcome (SUV ratio of myocardium to blood pool).

**FIGURE 7.**
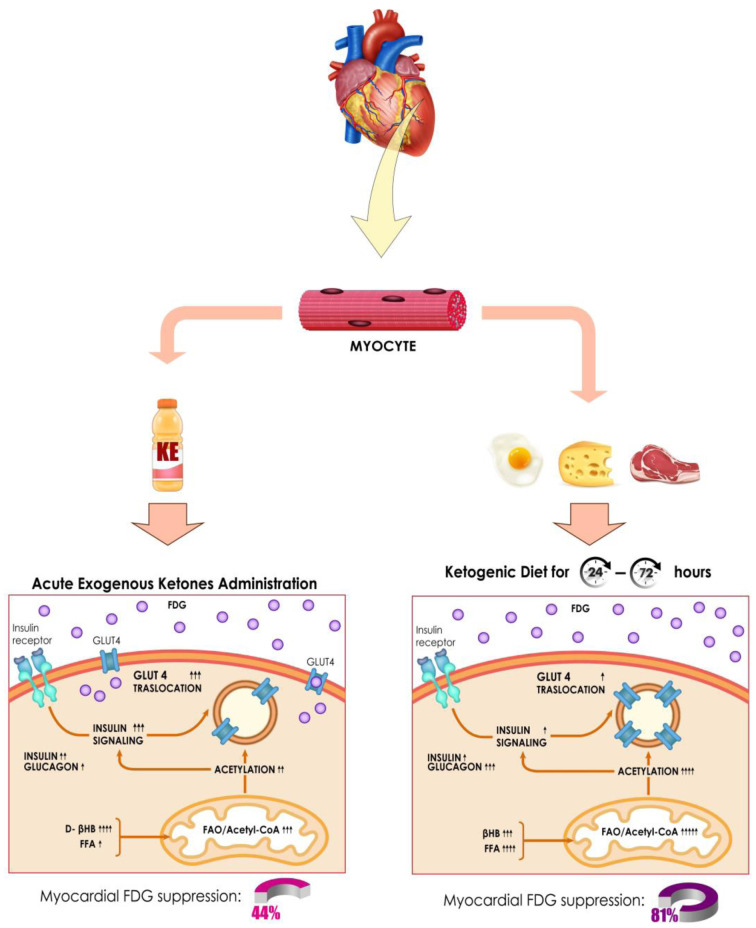
Proposed molecular mechanisms associated with myocardial ^18^F-FDG suppression between acute exogenous ketone administration (lower left) and endogenous ketosis induced by dietary modification (lower right). Posttranslational protein acetylation and attenuation of insulin signaling are both required mechanisms for successful inhibition of glucose transporter member 4 (GLUT4) translocation. Acute administration of KE drink leads to a severalfold increment in β-hydroxybutyrate (βHB) levels but also appears to decrease free fatty acid (FFA) levels, which may ultimately affect modulation of insulin signaling and process of acetylation. In contrast, although KD yields lower βHB levels after 24–72 h, it increases FFA and glucagon and lowers insulin levels on a sustained fashion, eventually augmenting fatty acid oxidation (FAO) over glucose oxidation (“Randle effect”), increasing protein acetylation, and limiting insulin signaling. These molecular differences in substrate handling may explain why myocardial ^18^F-FDG suppression rates were 44% for KE and 81% for KD. Adapted from “Insulin Mechanism,” by BioRender.com (2021 (*17*)).

Representative images further illustrating the correlation between common myocardial ^18^F-FDG uptake patterns and their respective BHB levels are shown in Figure 5. The relationships between BHB levels and glucose uptake using spline analyses are shown in Figures 6 and 7.

We also assessed the relationships between pre-KE drink BHB levels, post-KE drink BHB levels, and the difference between these levels with MGS. All 3 measurements were lower among those who failed MGS than those who appropriately suppressed (*P* < 0.05 for all comparisons) (Supplemental Table 3). All 3 measurements also significantly predicted MGS (AUROC 0.85, 95% CI 0.62–1.00; AUROC 0.85, 95% CI 0.67–1.00; AUROC 0.82, 95% CI 0.60–1.00, respectively).

Log BHB levels after the KE drink were significantly associated with MGS (standardized odds ratio 4.92, 95% CI 1.25–18.43, *P* = 0.022). Alternatively, log-transformed NEFA levels (standardized odds ratio 2.07, *P* = 0.16), insulin levels (standardized odds ratio 1.02, *P* = 0.97), and glucagon levels (standardized odds ratio 1.37, *P* = 0.51) were not predictive of MGS after the KE drink.

### Utility of Point-of-Care (POC) Ketone Testing to Predict MGS

To enhance clinical utility and provide a rapid assessment of ketosis before ^18^F-FDG PET, we also investigated POC ketone testing (paired testing available in 59 samples). Bland–Altman and scatter plots are shown in Supplemental Figures 4 and 5 comparing laboratory-derived BHB values and POC BHB values. The mean difference of laboratory- and POC-derived BHB levels was 0.04 (95% CI −0.88, 0.97 mmol/L), and correlation was strong (Spearman ρ 0.96, *P* < 0.001).

Since agreement was worst at the highest BHB levels, which were obtained after KE administration, we also assessed BHB levels obtained during the KD visits separately (as these lower values are encountered clinically with the KD preparation, *n* = 28 samples). The mean difference of laboratory- and POC-derived BHB levels was −0.02 (95% CI −0.29, 0.25 mmol/L), and correlation was strong (Spearman ρ 0.98, *P* < 0.001) (Supplemental Figs. 6 and 7).

POC BHB levels during the KD significantly predicted MGS (c-statistic 0.87, 95% CI 0.66–1.00) (Supplemental Figs. 8 and 9). Using POC BHB levels ≥ 0.6 mmol/L for predicting MGS correctly classified 89% of scans.

### Adverse Events

Mild (grade 1) adverse events occurred in 9 participants in the KE drink arm (these adverse events were mostly gastrointestinal [nausea or heartburn, *n* = 7] including 1 participant who experienced emesis, and 2 participants who reported a headache). In the KD arm, 1 participant reported presyncope (grade 1), and another participant reported back pain (grade 2), which led to study discontinuation.

## DISCUSSION

^18^F-FDG PET plays a critical role in assessing cardiovascular inflammation, infection, and tumors, yet adequate myocardial preparation to suppress physiologic glucose uptake remains a substantial diagnostic barrier, particularly as certain patterns of incomplete suppression (Fig. 5) can be indistinguishable from pathology. Such challenges may ultimately lead to misdiagnosis, inappropriate therapy, along with potentially harmful side effects, extra radiation exposure for repeated testing, and unnecessary costs to the patient and health-care system.

In KEETO-CROSS, an open-label, crossover trial of healthy participants, we found that a generalized strategy of KE was inferior to 24- and 72-h KD to achieve MGS. However, a few individuals who failed the KD were able to suppress with the KE drink. Importantly, however, we found that serum BHB levels during the KD robustly predicted MGS and identified potential thresholds that can be used upstream of ^18^F-FDG PET to predict MGS, thereby reducing false-positive or indeterminate scans. To facilitate clinical implementation, we tested the utility of a POC ketone meter that showed an ability to predict MGS similar to laboratory-based analysis. Thus, our findings support the continued use of the dietary modification through the KD rather than KE to achieve MGS, provide strong evidence for assessing BHB levels before ^18^F-FDG PET to ensure adequate dietary preparation, and demonstrate how a POC ketone device can be implemented to aid in rapid-decision making.

Despite several strategies to facilitate the myocardial “metabolic switch” to suppress physiologic glucose uptake, current MGS rates vary substantially, though much of these data are derived retrospectively and from convenience cohorts referred for clinical indications (*2*). The current trial was designed to address these challenges by investigating the use of an oral KE, which could facilitate same-day scans without patient preparation, in healthy volunteers. Endogenous and exogenous ketosis suppress myocardial glucose uptake in different ways (Fig. 7). Although endogenous ketosis has been standard-of-care for MGS, exogenous ketones also reduce myocardial glucose uptake, as shown in preclinical and clinical work (*11**,**12*). However, our results demonstrate that despite significant acute ketosis achieved, rates of suppression were inferior to the KD. We hypothesize several mechanisms for failure of the KE to uniformly suppress myocardial glucose uptake in these healthy volunteers. Posttranslational protein acetylation and attenuation of insulin signaling are necessary for the efficient inhibition of the glucose transporter member 4 (GLUT4) translocation (*13*) and thus ^18^F-FDG accumulation in myocytes. In this sense, acute exogenous ketone delivery may not engender effective substrate competition and a “Randle Effect” in the healthy heart given normal mitochondrial function (*7**,**14*). For example, post-KE drink NEFA levels were significantly lower than that achieved by KD. In this way, the KD may be more effective because it feeds both ketones and fatty acids for substrate competition, leading to increased fatty acid oxidation and protein acetylation. Second, failure of exogenous ketones could relate to increased insulin levels and, thus, signaling, an effect that can be counterproductive to achieving MGS. Although, we did not find a relationship between post-KE insulin levels and MGS. Third, the hemodynamic effect of exogenous ketones could theoretically contribute to increased myocardial glucose uptake, consistent with the vital sign changes in our study (*11*). Finally, the metabolic effect of exogenous ketone administration may be more dependent on the duration, rather than the peak levels, of ketosis before the “metabolic switch” occurs. In this sense, it remains to be proven whether extending the delay between KE and ^18^F-FDG administration would have led to improved suppression rates.

Notably, we found that ketone levels strongly predicted MGS. Broadly speaking, ketogenesis is a complex process that is influenced by hormonal signaling (including insulin and glucagon levels), transcriptional regulation, and posttranscriptional modification (*1*). As the end result of several biologic processes that reflect the milieu of glucose regulation, it is not surprising that ketone levels significantly relate to MGS. In our study, endogenous BHB levels had an AUROC of 0.88 for predicting MGS, and a cutoff of 0.58 mmol/L accurately classified MGS 92% of the time. This predictive ability of BHB was stronger than those with insulin, glucagon, glucose, and NEFA, consistent with other studies that have found modest predictive ability for these biomarkers (*15*), and clarifies that ketosis is a more potent driver, or predictive biomarker, of MGS than these other metabolic processes. Such potent prediction has clinical implications, as BHB levels can be routinely assessed before ^18^F-FDG PET to ensure adequate patient preparation. For instance, we found that 86% of subjects with BHB levels < 0.58 mmol/L failed to make the metabolic switch, and oftentimes, the patterns of incomplete suppression were indistinguishable from inflammation (Fig. 5). Consequently, if BHB levels are below this threshold, a longer duration of the KD (e.g., 72 h) can be undertaken with the visit rescheduled. Such an approach could considerably minimize false-positive or nondiagnostic scans, decreasing diagnostic uncertainty in clinical reading, inappropriate diagnoses, unnecessary costs, and radiation exposure from repeated scans. Alternatively, KE could be theoretically combined with the KD in participants below the threshold, since higher levels of ketosis after KE significantly predicted MGS. Moreover, POC ketone testing allows for rapid triage of patients and has already been implemented in other clinical settings (*16*). Such testing showed high agreement with laboratory-based analyses at levels encountered during KD and similarly predicted MGS. Given the reasonable costs of ketone meters, it may be possible for patients to monitor ketone levels remotely before ^18^F-FDG PET, potentially avoiding unnecessary travel and improving clinical throughput if levels predict inadequate preparation. Importantly, however, further studies in cohorts of patients with heart failure will be needed given relative myocardial glucose avidity in the failing heart (*1*).

There are some limitations. Although we chose to study KE against an active comparator (KD, which is standard-of-care), a placebo (fasting-only) arm would have provided firm conclusions on whether KE (and not just prolonged fasting) did suppress myocardial glucose uptake in some participants. However, an additional arm would have engendered greater radiation exposure against a weak comparator, and historical data on MGS are available for fasting-only patient preparations (*2*). In addition, our sample size is modest. Finally, our study design is open-label, which was limited by practicality of the dietary intervention, though endpoint adjudication is blinded. Strengths include a strict definition of complete MGS, crossover design, inclusion of extended-duration KD in addition to the gold-standard 24-h KD preparation, analysis of several relevant hormones and metabolites, and assessment of a POC device.

## CONCLUSION

A general strategy of exogenously administered KE was inferior to KD to achieve MGS. BHB levels strongly predicted MGS in healthy participants and can be clinically implemented before ^18^F-FDG PET to minimize false-positive or nondiagnostic scans, potentially decreasing inappropriate diagnoses, radiation exposure, and health-care system costs. POC ketone testing can provide rapid and accurate triage for adequate patient preparation for ^18^F-FDG PET. Further study to validate the diagnostic value of BHB levels in other patient cohorts, including older individuals, diabetics, and subjects with cardiomyopathies, is warranted.

## DISCLOSURE

Dr. Selvaraj receives research support from the Doris Duke Charitable Foundation (Physician Scientist Fellowship Award 2020061), the Measey Foundation, Institute for Translational Medicine and Therapeutics (Junior Investigator Preliminary/Feasibility Grant Program award and Translational Bio-Imaging Center award), and the American Society of Nuclear Cardiology (Institute for the Advancement of Nuclear Cardiology award). KEETO-CROSS was funded by the American Society of Nuclear Cardiology (ASNC) Institute for the Advancement of Nuclear Cardiology (IANC) award (to Svati H. Shah) and the Department of Radiology at the University of Pennsylvania. Funding for statistical support was provided by the Penn Cardiovascular Disease Fellowship Innovation Fund. Laboratory analyses were also supported in part by Public Health Services Research Grant P30 DK19525 (University of Pennsylvania Diabetes Research Center Radioimmunoassay and Biomarkers Core), who were not involved in the design and conduct of the study; collection, management, analysis, and interpretation of the data; preparation, review, or approval of the manuscript; and decision to submit the manuscript for publication. No other potential conflict of interest relevant to this article was reported.

## Notes

KEY POINTS **QUESTION:** Is exogenous ketone administration noninferior to the standard ketogenic diet to achieve myocardial glucose suppression, and can a serum biomarker predict myocardial glucose uptake status? **PERTINENT FINDINGS:** Exogenous ketone administration was inferior to the ketogenic diet to achieve myocardial glucose suppression. BHB levels strongly predicted the metabolic switch of glucose uptake in the heart of healthy volunteers. **IMPLICATIONS FOR PATIENT CARE:** BHB testing can provide rapid and accurate triage for adequate patient preparation for cardiovascular inflammation imaging with ^18^F-FDG PET.
